# Photocatalytic-ozonation process in oxytetracycline degradation in aqueous solution: composite characterization, optimization, energy consumption, and by-products

**DOI:** 10.1038/s41598-023-38309-0

**Published:** 2023-07-10

**Authors:** Jamal Mehralipour, Siamak Darvishali, Susan Bagheri, Majid Kermani

**Affiliations:** 1 Research Center for Environmental Health Technology, Iran University of Medical Sciences, Tehran, Iran; 2 Department of Environmental Health Engineering, School of Public Health, Iran University of Medical Sciences, Tehran, Iran

**Keywords:** Environmental sciences, Chemistry

## Abstract

In this research, we synthesized BiOI/NH_2_-MIL125(Ti) via solvo-thermal method to investigation of oxytetracycline (OTC) degradation in photocatalytic-ozonation process. The results of the XRD, FESEM, EDAX, FTIR, UV–Vis, TEM, XPS, and BET analyzes indicated that the catalyst BiOI/MOF was synthesized with excellent quality. Design of experiment (DOE), ANOVA statistical analysis, interaction of parameters and predicated optimum condition was done based on CCD. The effect of catalyst dose (0.25–0.5 mg/l), pH (4–8), reaction time (30–60 min) and O_3_ concentration (20–40 mN) at 10 mg/l of OTC on PCO/O_3_ process was optimized. Based on P-value and F-value coefficients (0.0001, 450.3 respectively) the model of OTC (F-value = 2451.04) and (P-value = 0.0001) coefficients, the model of COD removal was quadratic model. Under optimum condition pH 8.0, CD = 0.34 mg/l, RT = 56 min and O_3_ concentration = 28.7 mN, 96.2 and 77.2% of OTC and COD removed, respectively. The reduction of TOC was 64.2% in optimal conditions, which is less than the reduction of COD and OTC. The kinetics of reaction followed pseudo-first-order kinetic (R^2^ = 0.99). Synergistic effect coefficient was 1.31 that indicated ozonation, presence of catalyst and photolysis had a synergistic effect on OTC removal. The stability and reusability of the catalyst in six consecutive operating steps was acceptable and 7% efficiency decreased only. Cations (Mg^2+^, and Ca^2+^), SO_4_^2−^ had no influence on performing the process, but other anions, organic scavengers, and nitrogen gas, had an inhibitory effect. Finally, the OTC degradation probably pathway includes direct and indirect oxidation that decarboxylation, hydroxylation, demethylation and were the main mechanism in OTC degradation.

## Introduction

Research on the environment plays a crucial role in human health and development. The widespread of infections in recent decade will end up with the use of consumptions of medicinal compounds, especially antibiotics^1^. Environmental concerns have increased in recent years because of antimicrobial metabolites and their parents. Massive volumes of antibiotics are discharged to the environmental with the use of to human, animal, and aquaculture. It has been predicted that 100–200 thousand tons of antibiotics are consumed worldwide per year^2^. Human medicine, animal husbandry, and aquaculture use oxytetracycline (OTC) most frequently as a type of tetracycline antibiotic (TC)^3^. It is a complex, isoionic organic compound consisting of a sophisticated four-ring structure with multiple ionizable functional groups^4^. Because of confined absorption by humans and animals, about 27 to 75% of excess OTC as general structure is released into the environment^5^. Based on reports, OTC has been found in water, wastewater, soil, eggs, meat, and milk with variable concentrations ranged from ppb to ppm^6^. Until this point, Adsorption, Fenton regent, derivatives of Fenton oxidation, biological process, etc. have been used to remove and decompose OTC from aqueous^7–9^. As one of several degradation processes, photocatalytic ozonation (PCO/O_3_) is a subset of advanced oxidation processes (AOPs). It uses light, ozone molecules, and semiconductor catalysts in combination. Due to its attractive properties such as inexpensive, eco-friendliness, availability, suitable efficiency, and simple operation compared to other processes, it creates a fundamental function in the organic matter degradation^10^. It was shown that this integrated process enhances reactive oxygen species and subsequently enhances mineralization rates of resistant organic compounds, such as OTCs. The ozone molecule has just 2.07 V standard oxidation–reduction potential (ORP), while PCO/O_3_ process uses free oxidation radicals such as hydroxyl radical and reactive oxygen species (ROSs), that have higher than 2.0 eV ORP. In the photocatalytic process (PCO), the OTCs decomposition is restricted close the catalyst's surface, whilst in the integrated process, OTCs can be degraded to inorganic compounds such CO_2_ and H_2_O via ozone in solution^11^. The PCO/O_3_ process mechanism described by (Eqs. 1–12)^10^.

1 $$ {\text{Photo semiconductors}} + {\text{light }}\left( {{\text{UV}} - {\text{C}},\;{\text{A}}\;{\text{and}}\;{\text{visible}}} \right)\, \to \,\left( {{\text{h}}^{ + } + {\text{e}}^{ - } } \right)\;{\text{pairs}} $$

2 $$ {\text{O}}_{{3{\text{aq}}}} + {\text{e}} - \to {\text{O}}_{{3{\text{aq}}}}^{ \cdot - } $$

3 $$ {\text{O}}_{{3{\text{aq}}}}^{ \cdot - } + {\text{H}}^{ + } \to {\text{HO}}_{3}^{ \cdot - } $$

4 $$ {\text{HO}}_{3}^{ \cdot - } \to {\text{O}}_{2} + {\text{OH}}^{ \cdot } $$

5 $$ {\text{O}}_{{3{\text{aq}}}} + {\text{H}}_{2} {\text{O}} + {\text{light}} \to {\text{H}}_{2} {\text{O}}_{2} + {\text{O}}_{2} $$

6 $$ {\text{H}}_{2} {\text{O}}_{2} + {\text{light}} \to 2\;{\text{OH}}^{ \cdot } $$

7 $$ {\text{H}}_{2} {\text{O}}_{2} + {\text{O}}_{{3{\text{aq}}}} \to 2\;{\text{OH}}^{ \cdot } + {\text{O}}_{{3{\text{aq}}}}^{ \cdot - } $$

8 $$ {\text{H}}_{2} {\text{O}}_{2} \to {\text{OH}}_{2}^{ - } + {\text{H}}^{ + } $$

9 $$ {\text{O}}_{{3{\text{aq}}}} + {\text{OH}}_{2}^{ - } \to {\text{O}}_{{3{\text{aq}}}}^{ \cdot - } + {\text{OH}}_{2}^{ \cdot } $$

10 $$ {\text{OH}}_{2}^{ \cdot } \leftrightarrow {\text{O}}_{2}^{ \cdot - } + {\text{ H}}^{ + } $$

11 $$ {\text{O}}_{{3{\text{aq}}}} + {\text{O}}_{2}^{ \cdot - } \to {\text{O}}_{{3{\text{aq}}}}^{ \cdot - } + {\text{O}}_{2} $$

12 $$ {\text{Free}}\;{\text{radicals}} + {\text{OTCs}} \to {\text{CO}}_{2} + {\text{H}}_{2} {\text{O}} + {\text{non - toxic}}\;{\text{inorganic}}\;{\text{compounds}} $$

At the begging of the reaction chain (Eq. 1), UV light irradiates cause of the production of electrons and holes. Based on (Eq. 2) reaction, ozone molecule uses of photo-generated electron to generate O_3_^**·**−^. The oxygen captures electrons form HO_2_^**·**^/O_2_^**·**^, that could react with ozone and generate O_3_^**·**−^. According to (Eq. 3), O_3_^**·**−^ reacts via H^+^ to be transformed into HO_3_^**·**−^ and OH^**·**^ (Eq. 4). Based on (Eq. 5), Hydrogen peroxide (H_2_O_2_) generated that produces OH^**·**^ and O_3_^**·**−^ based on subsequent reactions. Newly, study about PCO/O_3_ process focalized in presentation of novel semiconductors. Some semiconductors like metal–organic framework (MOFs), g-C_3_N_4_, and slim band-gap oxide of metals families were used to expand the response spectrum of light until visible range^12^. The classic semiconductors like TiO_2_ and ZnO activated via UV-C (under 300 nm wavelength that is only 4% of sunlight spectrum)^13^. Recently, a growing attraction in MOFs has focused on photocatalysis for the photo-driven removal of pollutants^14^. There have been a variety of applications of them in recent studies, such as catalysis, chemical sensing, and adsorption^15^. Various MOF-based photocatalysis have recently been developed in environmental studies. The Ti-based amino-functioned MOF (NH_2_-MIL125 (Ti)) was chosen for some advantages, including non-toxicity, being cheap, photo/water stability, and visible-light absorption^16^. The BiOI/NH_2_-MIL125(Ti) catalyst was prepared to make a synergistic effect with outstanding photocatalytic activity^17^_._ Bismuth-based semiconductors as a novel form of nonpoisonous, consistent photocatalysis via high visible light absorbency are worthwhile, environment-friendly, and stable compounds^18^. Among these, BiOI has shown impressive photocatalytic activity due to its appropriate band gap (BG = 1.62–1.93 eV), exclusive layered structure, and Special electrical features. The high rate of recombination of BiOI restricts its practical application. It has been proposed that heterojunctions can overcome this disadvantage by increasing charge separation and reducing the photo-generated electrons and hole recombination^19^. One way to improve the efficiency of photocatalysis is to create a heterojunction structure. This method reduces the energy of the band-gap between the conduction band and the valence band, and as a result, the photocatalyst is activated in the range of visible and UV-A light. Also, the structure of catalyst BiOI/NH_2_-MIL125(Ti) is such that it overcomes the limitations of structures BiOI and NH_2_-MIL125(Ti) and finally becomes a catalyst with proper efficiency in the PCO/O_3_ process^20^. Alexsandra Valério et al., an investigation of the photocatalysis and ozonation process on the tetracycline degradation. In this study, the total organic carbon (TOC) removal reached above 90% after 3 h. Compared to the sum of the separated processes, the kinetics of reaction increase by 20%^21^. In this current study, for the first time, BiOI/NH_2_-MIL125(Ti) was used as a catalyst in the PCO/O_3_ process for the decomposition of OTC. This catalyst possesses a remarkable capacity to generate active free radicals because of its unique characteristics. Water and wastewater quality is degraded by synthetic organic compounds such as OTC due to their benzene rings and high carbon content. To assess the effectiveness of the PCO/O_3_ process in improving the quality of wastewater, besides examining the removal of OTC, chemical oxygen demand (COD) and TOC were also investigated. The principal aims of the current study were to synthesis of BiOI/NH_2_-MIL125(Ti) (in short BiOI/MOF) and optimization of parameters of PCO/O_3_ process for the degradation of OTC and COD. In a supplementary study, we investigated TOC decreasing, reaction kinetics, mechanisms that work together synergistically, catalyst's stability, organic co-existing and organic radical scavenger's effects, energy consumption and probably reaction path-way and by-products in PCO/O_3_ process.

## Method and materials

### Materials and reagents

All solvents and reagents bought from reputable companies (Merck, Sigma-Aldrich, and Samchun). Ethylene glycol [(CH_2_OH)_2_], potassium iodide [KI], bismuth nitrate pentahydrate [Bi (NO_3_)_3_·5H_2_O], *N*,*N*-dimethyl formamide [C_3_H_7_NO], tetra butyl titanate [C_16_H_36_O_4_Ti], 2-amino terephthalic acid [C_8_H_7_NO_4_], Oxytetracycline [C_22_H_24_N_2_O_9_], sodium hydroxide [NaOH], sodium bicarbonate [NaHCO_3_], sodium nitrate [NaNO_3_], oxalic acid [C_2_H_2_O_4_], acetonitrile [C_2_H_3_N], and methanol [CH_3_OH]. De-ionized water (16.25 MΩ/cm, Milli-Q purification system model: MILLI-Q® HX 7000 SD) was used for preparing solutions.

### Catalyst fabrication

#### MOF (NH_2_-MIL125 (Ti)) fabrication

Based on the literature, solvo-thermal method with some changes in the amount of materials was used to NH_2_-MIL-125 (Ti) fabrication^22,23^, A solution containing 33 ml of *N*,*N*-dimethyl formamide (DMF) and 4 ml methanol containing 1.39 g of 2-amino terephthalic acid and 1.915 ml of tetra butyl titanate was mixed for 25 min in an ultrasonic bathroom at room temperature. A Teflon-lined autoclave was used to maintain the mixture solution at 180 °C for 60 h. A final step was to filter away the formed suspension and thoroughly rinse with methanol and DMF several times, and then dry it at 75 °C in an electric furnace.

#### BiOI-MOF (BiOI/NH_2_-MIL125(Ti)) fabrication

Based on the literature, BiOI/MOF was fabricated with a few changes in the amount of materials^22^. A suspension of MOF (2 g) was ultrasonically dissolved in 36 cc of deionized water with KI (0.19 g) for 20 min. Incorporated in 13 ml of ethylene glycol, the solution was gently diluted with Bi (NO_3_)_3_·5H_2_O (0.97 g). In a water bath at around 80 °C, the suspension was stirred vigorously for 30 min. During refinement, the catalyst was filtered, washed three times with ethanol and distilled water, and dried at 80 °C.

#### BiOI fabrication

To investigation and comparison of physical, chemical, and other properties of MOF, BiOI, and BiOI/MOF, In the absence of MOF in the solution, pure BiOI was synthesized.

#### Characterization

A FT-IR spectrophotometer was used to examine the chemical structures of BiOI, MOF, and BiOI-MOF (Sipotlight 220i FT-IR Microscopy Systems; 4000–400 cm^−1^). X-ray diffraction (XRD) analysis of XRD diffractometer Rigaku/ZSX Primus 500; radiations source: Cu Kα [(λ = 1.54056 Å) monochromatic incident beam between 5° to 80° with the step interval of 0.02°, and rate of 0.05°/s] was assessed to survey the crystal structure of the samples. Scherrer's equation (Eq. 13) was used to calculate the average samples sizes^24^,

13 $$D=\frac{(K.\lambda )}{(\beta .cos\theta )}$$

where D represents the crystal samples size, λ is the angle of diffraction, K represents the dimensionless constant, β is FWHM (full width half maximum) of the diffraction peak and θ is the diffraction angle and θ is the diffraction angle.

The optic properties and structural features were examined using UV–Visible spectrum (UV–Vis DRS) was recorded by Agilent Cary 60 spectrophotometer. Tauc equations (Eqs. 14, 15) were used to determination of band-gap energy^25^.

14 $$\alpha hv={A\left(hv-Eg\right)}^{1/2}$$

15 $$\alpha hv={A\left(hv-Eg\right)}^{2}$$

where, α is the coefficient of absorption, v is frequency of light, A is a proportionality constant, h is Planck's constant, and E_g_ is the band-gap. The band-gap energy (direct band-gap semiconductor) of BiOI is calculated using Eq. (14) while that of MOF (indirect band-gap semiconductor) is calculated using Eq. (15). The morphology of samples was observed by FE-SEM (UN41219SEM) under vacuum conditions of ≥ 1.2 × 10^−4^ mbar. An analysis of elemental mapping and purity was performed on samples using energy dispersive spectroscopy (EDS). Shimadzu JEM-1200 EX with 100 kV stimulating voltage was used to obtain transmission electron microscopy image (TEM). Nitrogen adsorption at 77 K was used to determine the volume, pore size, and surface area distribution of BiOI/MOF. In situ degassing of samples was conducted for 12 h at 200 °C under vacuum. Brunauer–Emmet–Teller (BET) applied to calculate surface areas according to the linear relationship between p/p_0_ and surface areas. An ESCALAB250XI electron spectrometer was used to analyze the XPS characterizations.

### Photocatalytic ozonation process set up

**Figure 1:**
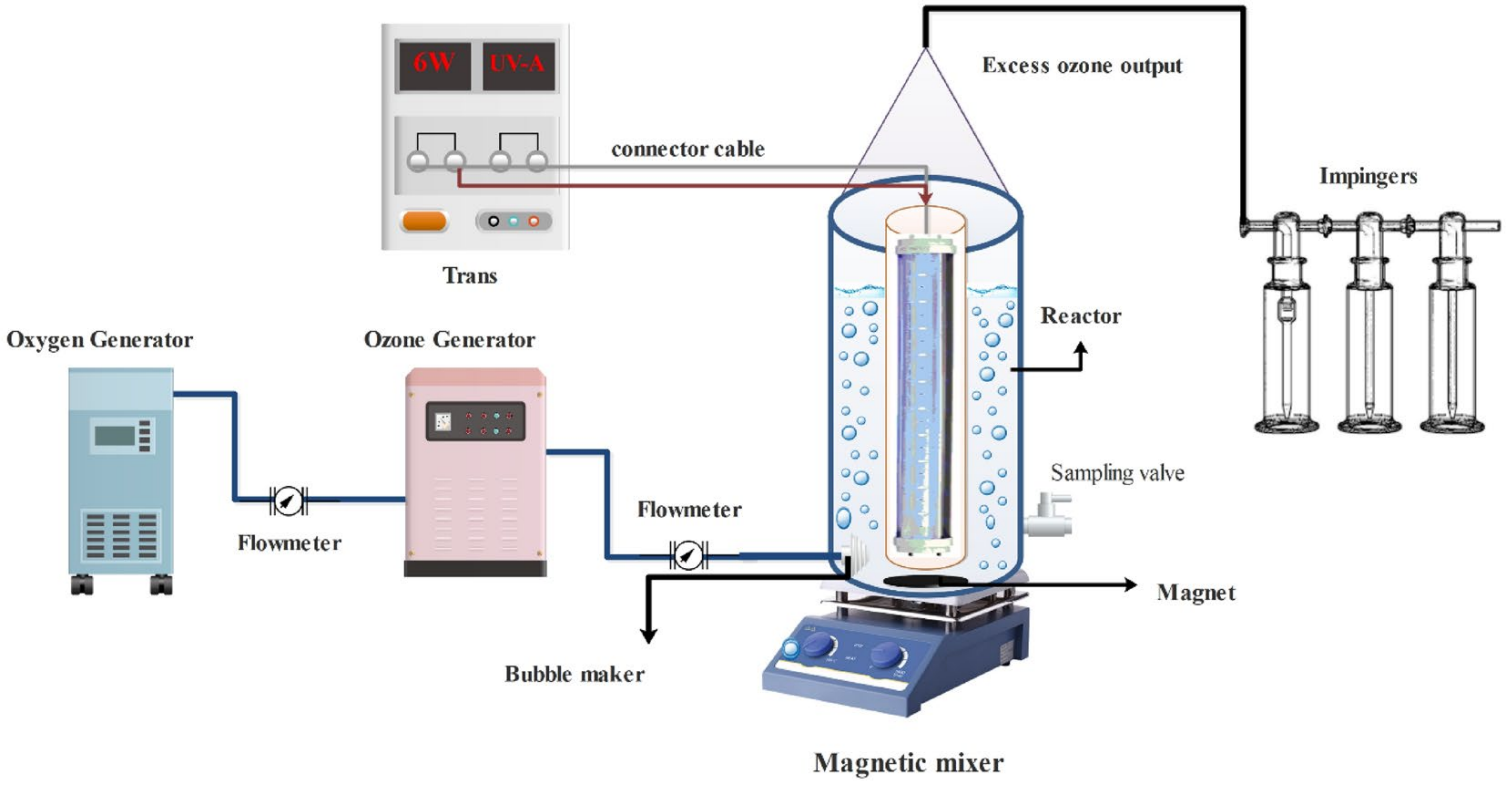
Schematic of PCO/O_3_ process.

PCO/O_3_ process runs were done in a 600-ml cylindrical unit with D = 10 cm and h = 20 cm dimensions. The quartz sheath with dimensions 4.5 × 16 cm was installed horizontally in the middle of the reactor. The UPVC box with 39.5 × 41.5 × 30 cm was used as a pilot unit (Fig. 1). A batch system was used to perform the runs at ambient temperature (25 ± 2 °C). 0.1 g of OTC was dissolved into deionized-water for preparation of stock solution (100 mg/l). The UV-A (Low-pressure, *λ* = 385 nm maximum wavelength range, and 6 W) was installed in the middle of reactor, with 15 cm length (TUV-PLL6W-UVC model, PHILIPS). By passing oxygen-feed gas through an ozone generator (O180F/DST, Canada), a steady flow of ozone gas was introduced into the reaction media by a micro-diffuser. O_3_ concentration adjusting via flow rate of feed oxygen. According to 2350 E method (iodometric) of the standard methods for the examination of water and wastewater, ozone generator capacity and ozone concentration in the reactor and output were determined^26^. The contents of the reactor during the PCO/O_3_ process was mixed by a magnetic stirrer. To measure OTC, 10 ml of each reaction mixture were filtered with 0.22 m syringe filters after being taken from the reactor and injected into high-performance liquid chromatography (HPLC). As the eventual results, mean values were calculated from three repetitions of each test. OTC, COD, and TOC degradation percentages in the PCO/O_3_ process were calculated using the following (Eq. 16)^24^.

16 $$ {\text{Efficency}}\;{\text{removal}}\;{\text{(\% )}} = \left[ {\frac{{{\text{C}}_{{0}} - C_{t} }}{{C_{0} }}} \right] \times 100 $$

where, C_o_ and C_t_ are the concentrations of initial and residual OTC, COD, and TOC (mg/l) at time t, respectively.

### Optimization of PCO/O_3_ variables based on central composite design

The undertaking of PCO/O_3_ process for OTC, COD, and TOC removal was optimized at 10 mg/l OTC as an initial concentration via central composite design (CCD) as an approach in the response surface method. The range of variables is illustrated in Table 1.

**Table 1:** The Range of influence factors on PCO/O3 process performance.

Variables	Name	Units	Minimum	Maximum	Coded. low	Coded. high	Mean	Std. dev.
A	pH		2	10	− 1 ↔ 4	+ 1 ↔ 8	6	1.82
B	Dose of MOFs	mg/L	0.125	0.625	− 1 ↔ 0.25	+ 1 ↔ 0.5	0.375	0.1137
C	Reaction time	Min	15	75	− 1 ↔ 30	+ 1 ↔ 60	45	13.65
D	O_3_ Concentration	mN	10	50	− 1 ↔ 20	+ 1 ↔ 40	30	9.10

Table 2 shows the number of 30 runs intended for the removal of OTC, COD, and TOC based on the CCD design.

**Table 2:** Runs of PCO/O3 process into OTC, COD, and TOC removal.

Run	Factor. 1	Factor. 2	Factor. 3	Factor. 4	Response. 1		Response. 2	
	A:pH	B: Dose of MOFs	C: Reaction time	D: O_3_ Concentration	OTC Observed removal	OTC predicated removal	COD Observed removal	COD predicated removal
Unit		mg/L	min	mN	%			%
1	8	0.25	60	40	85	84.9	67.83	68.05
2	6	0.375	45	30	90	89.8	70.16	72.3
3	8	0.5	60	20	91.7	93.77	74.16	73.74
4	8	0.5	30	20	74.3	73.15	57.2	58.95
5	6	0.375	45	30	90	89.8	70.16	72.3
6	4	0.25	30	40	53.7	53.25	40.5	41.44
7	4	0.5	30	40	65.3	65.32	51.37	51.30
8	6	0.375	45	30	90	90	72	72.3
9	4	0.5	60	40	77.2	77.58	61.3	61.42
10	6	0.375	75	30	95	96.14	76.79	76.55
11	6	0.375	15	30	61.1	60.3	46.62	47.73
12	4	0.25	60	40	68.8	68.47	53.7	54.28
13	10	0.375	45	30	93	93.1	74.45	74.85
14	8	0.25	60	20	88	85.9	74.45	70.6
15	4	0.5	60	20	80	79.28	68.54	63.8
16	8	0.5	60	40	95	94.57	62.04	76.55
17	6	0.375	45	30	90	89.8	75.2	72.3
18	4	0.5	30	20	58.6	59.52	46.87	45.61
19	8	0.25	30	40	68.6	68.83	45.83	54.11
20	4	0.25	60	20	71.9	71.97	56.16	56.91
21	4	0.25	30	20	49.3	49.25	37.7	37.70
22	6	0.375	45	30	90	89.8	73.58	72.3
23	2	0.375	45	30	62.8	63.04	48.95	49.18
24	6	0.375	45	10	60	60.82	46.29	46.8
25	8	0.25	30	20	62.2	62.3	48.33	48.67
26	6	0.375	45	30	90	89.8	73.58	72.3
27	6	0.375	45	50	65.5	65.62	51.12	51.47
28	6	0.625	45	30	83	82.84	65.4	66.35
29	6	0.125	45	30	62.4	62.9	48.95	48.84
30	8	0.5	30	40	80.5	81.45	64.5	64.22

The empirical second-order polynomial model of OTC removal via PCO/O 3 was described by Eq. (17)^27^:

17 $$ Y = \beta_{0} + \sum\limits_{i = 1}^{k} {\beta_{i} } x_{i} + \sum\limits_{i = 1}^{k} {\beta_{ii} } x_{i}^{2} + \sum\limits_{i = 1}^{k - 1} {\sum\limits_{j = i + 1}^{k} {\beta_{ij} } } x_{i} x_{j} + \varepsilon $$

Here, Y is OTC decrease (%),β_0_ is the intercept, β_i_, β_ii_, and β_ij_ are the linear, quadratic, and interaction effect coefficients of variables respectively, x_i_ and x_j_ are coded testing classes of the variables, k is the number of the independent variables, and e is the residual error. A coded value for each variable was calculated using Eq. (18), to enable comparison between factors with different units.

18 $$ x_{i} = \frac{{X_{i} - X_{0} }}{\Delta x} $$

where, the coded value of the variable is Xi, *∆x* which is the difference between the high and low values of the variable, X_0_ represents the low value of the variable. Using P-values and F-values, analysis of variance (ANOVA) was performed to determine the interaction between response and factors. R^2^ and R^2^_adj_ are correlation coefficients. In addition, R^2^ predicts were used. Then, In the optimal condition, organic, and inorganic radical scavengers effect, degradation kinetic based in difference OTC concentration, the synergistic effect on individual and combined processes (photolysis, simple ozonation, adsorption in dark mood, UV-O_3,_ photocatalysis, and PCO/O_3_ process, the light source type (UV-C, UV-A, and visible light) effect, degradation pathway, and intermediates production, changes in wavelength scanning in during the PCO/O_3_ process, and calculation of energy consumption were performed.

### Analytical methods

Each sample was collected after the runs and processed through a 0.45 m micro-filter before injection into HPLC. The amount of OTC was measured using a HPLC (Ciciel company, pump:120 mm × 240 mm × 440 mm, pressure: 40 MPa, flow rate setting range: 0.001–20 ml/min, solvent delivery method: parallel-type double plunger, plunger capacity: 10 μl) equipped with a UV–Vis detector (C18 column, 250 mm × 4.6 mm, with 5 μm particle size, pore size: 12 nm, surface area: 410 m^2^/g, carbon loading: 20%, pore volume: 1.25 ml/g, pH range 2–7.5, bonding type: monomeric) and a column. Temperatures of 4–25 °C and 353 nm, respectively, were kept constant for UV detection and column hold. Mobile phase consisted of 95:5 (v/v) mixture of acetonitrile and ultra-pure water (0.05% ammonia) at 1.0 ml/min flow rate. A 10 μl of OTC solution was injected at a retention time of 12 min. The TOC analysis was performed with Analytikjena's multi C/N 3100 TOC analyzer. As described in the standard method, COD was measured using the titrimetric approach (5220-C; closed-reflux)^28^. Residual catalyst and ROS were two interferers in COD test. So, the COD samples were initially passed through a 0.22 µ PTFE filter. Due to the rapid rate of decay, ROS are rapidly eliminated. The decay of the intermediates was studied by gas chromatography/mass spectrometry (GC–MS) (Agilent 7890A, California, USA). For the gas chromatography system, DB-5MS columns (30 m 0.5 lm film thickness) were used with high-purity (99.99%) helium as the carrier gas flowing at 1.0 ml/min. For temperature, the column was maintained at 35 °C for 1 min, increased to 300 °C at 7.0 °C/min, and held at 300 °C for 1 min. 10 cc of the sample was injected, with the injector and detector both set at 280 °C.

### Supplementary studies

#### Kinetics of reaction and synergist effect

Degradation of OTC in the optimum condition of PCO/O_3_ process described by the first-order reaction (Eq. 19)^29^.

19 $$ \ln (C_{t} /C_{0} ) = - k_{ppa} t $$

where, *C*_*t*_ is residual and *C*_*0*_ is initial OTC concentrations (mg/l), *t* is the reaction time (min) and *k*_*app*_ is the rate constant (1/min).

The synergist effect of single mechanism such as photolysis, simple ozonation, catalytic ozonation, photocatalytic, and adsorption were investigated in an optimum condition of parameters. The synergist effect calculated via (Eq. 20)^30^.

20 $$\mathrm{Synergist \, effect }=\frac{Performance \, of \, PCO/O3 \, process(\%)}{Adsorption+photolysis+simple \, ozonation (\%)}$$

#### Stability of catalyst, effect of radical scavengers

The stability of catalyst in optimum condition of PCO/O_3_ process was tested in five runs. Also, the effect of inorganic and organic radical scavengers investigated in optimum condition via tert-butyl alcohol (*t*-BuOH or TBA), N_2_ gas, Oxalic Acid, NO_3_^−^, Cl^−^, SO_4_^2−^, Mg^2+^, Ca^2+^ and HCO_3_^−^.

#### Electrical energy consumption

The electrical energy consumption (EEC) of PCO/O_3_ process under optimum condition calculated by (Eq. 21)^31^.

21 $$EEC=\frac{38.4*P}{V*Kobs}$$

where P (kWh) is the consumption of power and V (l) is the volume of the treated solution.

#### Mineralization, by products, intermediates, and degradation mechanism studies

In this part of the study, we investigated mineralization, by products, intermediates, and degradation mechanism of OTC in optimum condition of PCO/O_3_ process.

## Results and discussion

### Characters of MOF

#### XRD

**Figure 2:**
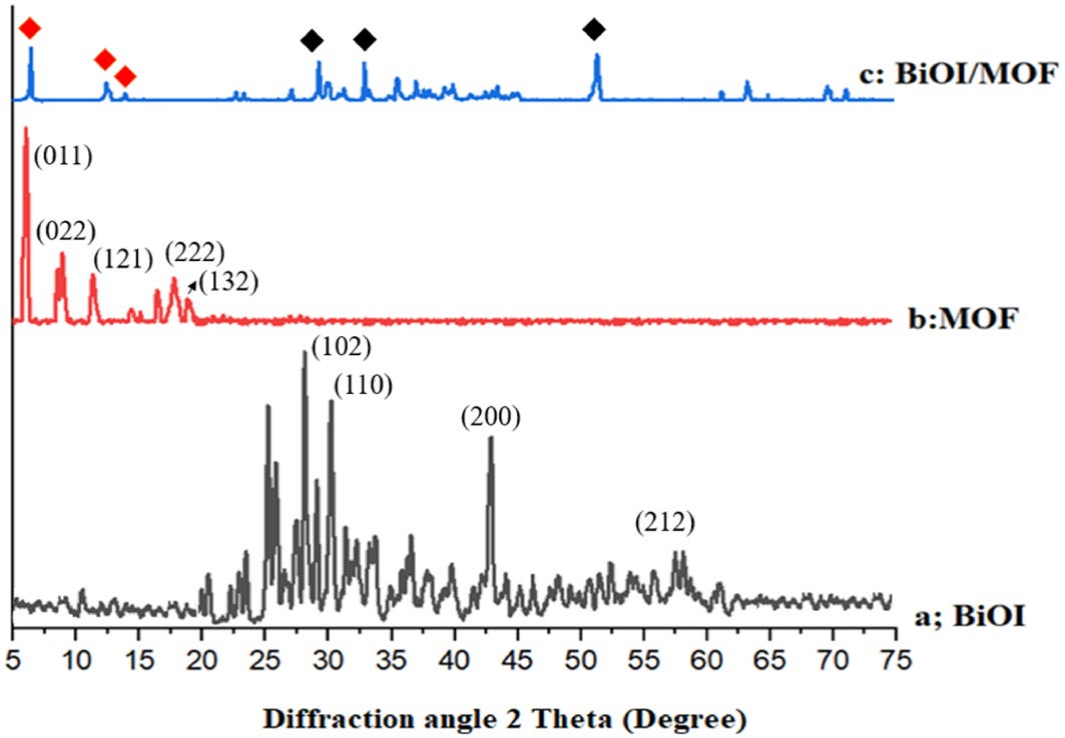
XRD patterns of BiOI, MOF, BiOI-MOF.

The crystalline structure of the prepared BiOI, NH_2_-MIL125, and BiOI-MOF were analyzed through powder XRD patterns and the obtained finding are showed in (Fig. 2). The four major peaks in BiOI's XRD pattern (Fig. 2a) are (102), (110), (200), and (212) respectively. A tetragonal phase was observed in all diffraction peaks for synthesized BiOI (JCPDS no. 10-0445). High crystallinity is clear from the strong and sharp peaks. According to Han and coworkers' study, XRD analysis was similar^32^. MOF crystal formation is shown in (Fig. 2b). In the standard pattern, MOF peaks were observed at 6.7°, 9.8°, 11.9°, 15.06°, 16.6°, 17.9°, 19.07°, and 19.6°. It is clear from XRD patterns that orthorhombic phase correlates well with lattice parameters a = 15.03 Å, b = 6.2 Å, and c = 19.15 Å. By BDC and NH_2_BDC as a linker, three-dimensional pores are produced in a chine of corner-sharing TiO_6_. A similar result was reported by Ovisi and coworkers^33^. As shown in Fig. 2c, the BiOI-MOF structure also exhibits some peaks in the BiOI and MOF structures. According to the MOF, the prominent peak lies two degrees below 10 degrees. It appears at 25–35 degrees for BiOI peaks. Peaks' positions and intensity both change significantly. XRD results showed similarity in Du and coworkers' study (36). Based on Scherrer's equation (Eq. 13), the crystal size of BiOI, MOF, BiOI-MOF samples are 26/5, 47/8 and 37/1 nm.

#### FT-IR

**Figure 3:**
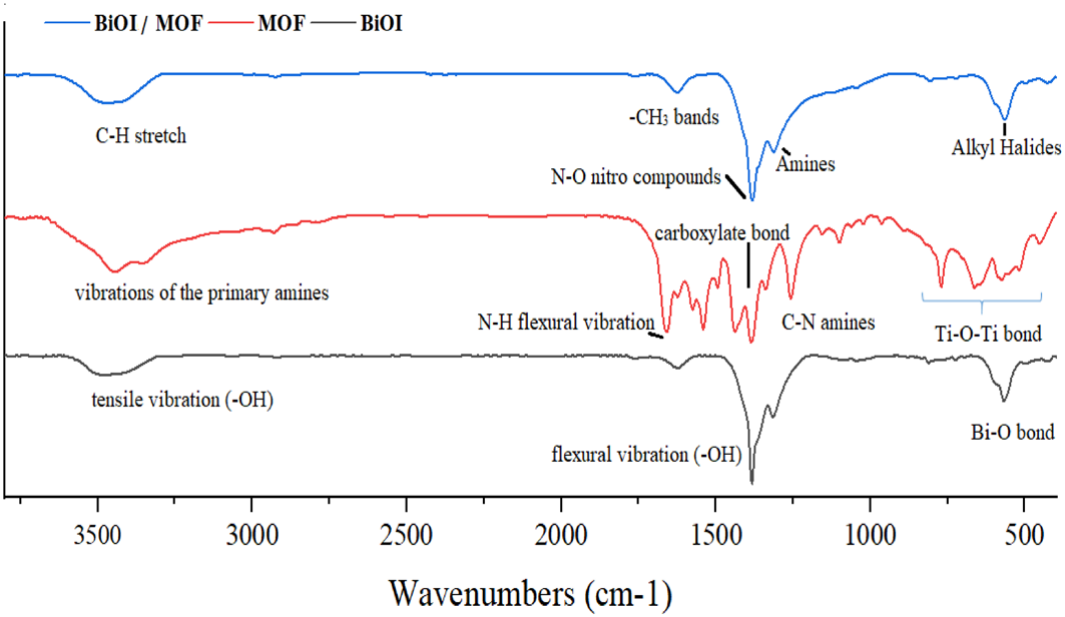
FTIR spectrum of BiOI, MOF, BiOI-MOF.

In order to investigate the organic groups of samples, the FTIR spectrum was conducted (Fig. 3).

Five peaks at 571.7, 1309.5, 1382.2, 1627.4, and 3529.1 cm^−1^ position identified in BiOI (Fig. 3a). A_2_u type Bi-O bond has a symmetrical vibration at 571.7 cm^−1^. BiOI exhibits strong adsorption at 1300 cm^−1^ to 1700 cm^−1^, as well as a large peak at 3175 cm^−1^ to 3470 cm^−1^ as a result of flexural (-OH) and tensile vibrations (–OH) of free water molecules on the surface of the BiOI, respectively. Similar results reported by Ai and coworkers^34^. The peaks between 500 and 1500 cm^−1^ for MOF shows characteristics of organic compounds (Fig. 3b). Typical tensile strengths of aromatic N–H flexural vibrations and C-N amines can be attributed to the peaks at 1255 cm^−1^ and 1622 cm^−1^, respectively. The carboxylate bond is indicated by two obvious peaks at 1535 cm^−1^ and 1433 cm^−1^. In addition, the absorption peaks around 450 cm^−1^ are associated with the typical vibration of Ti–O-Ti bonds. Primary amines also exhibited broad peaks at 4000 cm^−1^ for both symmetric and asymmetric tensile vibrations. Among the major peaks reported by Zhao and coworkers, there were 773, 1258, 1385, 1539, 1662, 2524, 3059, 3348, and 3450 cm^−1^^35^. BiOI-MOF FT-IR spectrum was illustrated in (Fig. 3 c). Peaks were observed at 565.5, 807, 1313, 1382, 1624, and 3464 cm^−1^. Amines, Alkyl Halides, –CH_3_ bands, N–O Nitro compounds, C–H stretch, and C–H bands can be found on these peaks. A study conducted by Han et al., and another by Du et al., confirmed the heterojunction between BiOI at NH_2_ with NH_2_-MIL125 (Ti)^22,36^.

#### Morphology analysis

**Figure 4:**
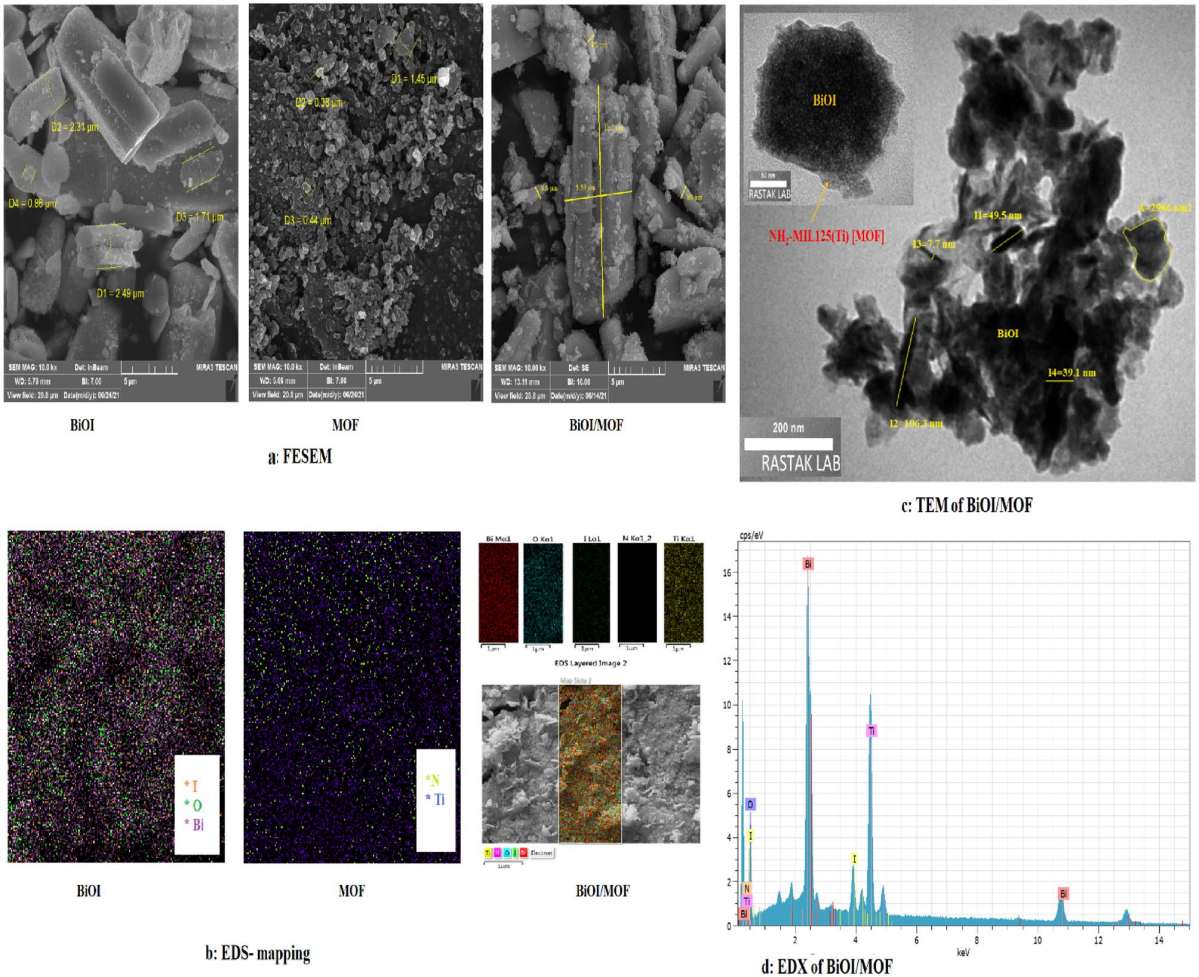
SEM of (**a**) BiOI, (**b**) MOF, (**c**) BiOI-MOF, (**d**) EDX of BiOI-MOF, (**e**) TEM of BiOI-MOF.

Samples are analyzed by FESEM, EDX, EDS mapping and TEM (Fig. 4). FESEM images of BiOI, MOF, and BIOI/MOF was illustrated in (Fig. 4a). BiOI FESEM image shows the typical morphology that is thickness plate structures. It is relatively long to wide for a synthesized structure. An alternative solvent was used in Arumugam and coworkers' study. A 2D square-like nano-sheet morphology is observed with water (H_2_O-BiOI) as a solvent^37^. Synthesis methods, chemicals, and solvents used affect the morphology of BiOI. The synthesized MOF is thin and disk-like. A MOF's structure changes when its organic-metal ratio changes. Research has shown that MOF structures change from rectangular to round as organic linkers increase. The nanoplate morphology of NH_2_-MIL125(Ti) was uniform in Zhang and coworkers' study^38^. BiOI-MOF is shown as a rod containing fine particles. The morphology of BiOI/MOF was similar in Du and coworkers' study^17^. It can be seen from the TEM image (Fig. 4c) that the NH_2_-MIL125(Ti) composite has been distributed successfully on the surface of BiOI, forming a core–shell structure. In the (Fig. 4c), shows the results of EDS-mapping. The results show that the distribution of elements on the surface of all three structures is heterogeneous. Also, (Fig. 4d) shows EDX image of BiOI/MOF. Samples show different weight ratios for each structure's fundamental elements.

#### BET analysis

**Figure 5:**
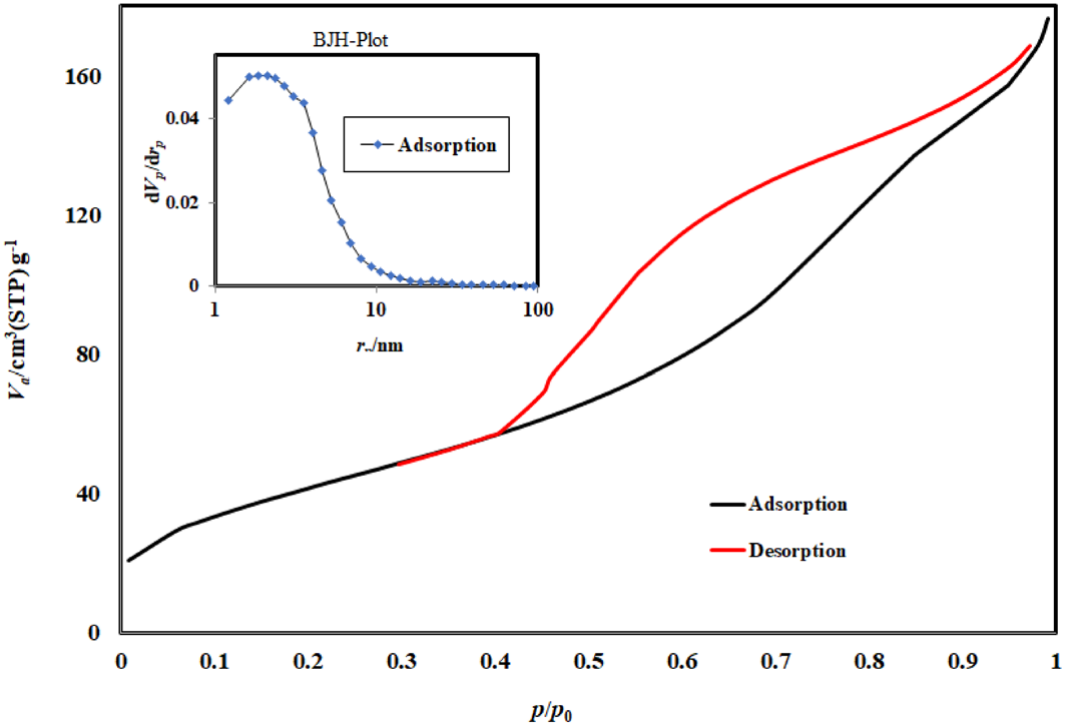
Adsorption/Desorption pilot of BiOI-MOF.

BiOI/MOF, nitrogen adsorption–desorption isotherms are shown in (Fig. 5). Relative pressure affects adsorption and desorption rate. In view of the curved shape, the hysteresis belongs to category H_3_ and type IV. A non-hard cavity with an incised shape has this type of hysteresis. The tensile strength effect, also known as the H_3_ hysteresis repulsion branch, has a steep slope. By N_2_ adsorption–desorption isotherm and BJH, the precursor's BET area of surface and pore volume were determined to be 947.85 m^2^g and 16.27 cm^3^/g, respectively.

#### UV–Vis analysis

**Figure 6:**
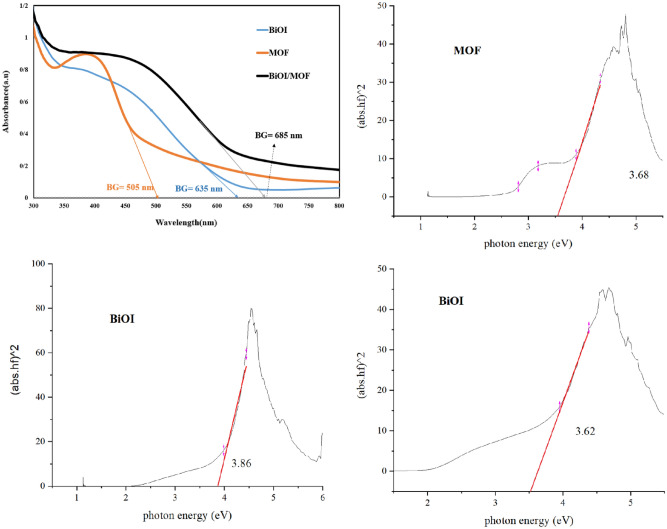
(**a**) UV–Vis and, (**b**–**d**) DRS analysis of BiOI, MOF, and BiOI-MOF.

Samples UV–Vis spectrum shown in (Fig. 6). The band edge of the BiOI, MOF and BiOI-MOF calculated at 635, 505 and 685 nm, respectively. The results show that the catalyst BiOI/MOF has a larger band edge compared to pure BiOI and MOF. The band edge is in the visible light range, indicating that a visible light source can also activate the catalyst. Also, DRS analysis showed that the BiOI/MOF catalyst has a shorter band gap than BiOI and MOF and requires less energy for activation. In our pervious study, similar results were reported^26^.

#### XPS analysis

**Figure 7:**
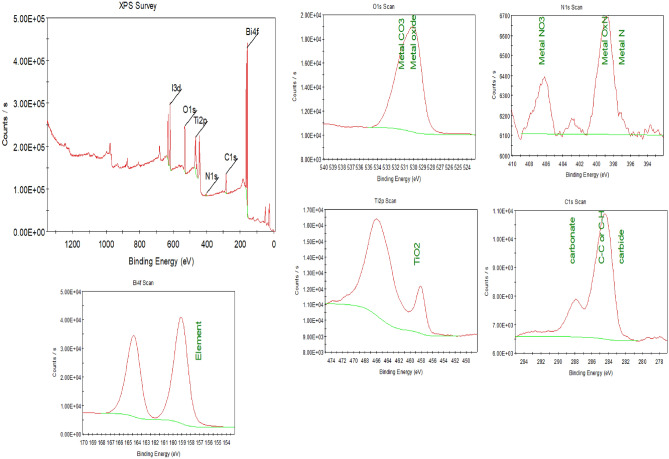
XPS spectra of BiOI-MOF.

The chemical state and surface composition of BiOI-MOF were determined using XPS (Fig. 7). The XPS spectrum confirms elements O, Ti, C, I, and Bi. The composition purity was verified by BiOI and NH_2_-MIL125(Ti). An overview of BiOI-MOF structure elements is presented in Table 3. Similar results were obtained by Jiang and coworkers^18^.

**Table 3:** Characteristics and quantities of elements in BiOI-MOF.

Name	Peak BE	FWHM eV	Area (P) CPS.eV	Weight %
I3d	619.29	2.55	690,831.13	12.51
Bi4f.	159.35	1.70	1,772,170.25	42.31
O1s	531.02	3.44	393,125.12	12.28
C1s	285.07	3.11	169,423.64	9.61
Ti2p	466.05	5.25	563,521.87	22.31
N1s	399.61	1.19	22,816.83	0.97

### Photocatalytic-ozonation activity

#### CCD design, fit, and statistical analysis

The statistical analysis of the OTC, and COD results provided the basis for response surface method (RSM) development and finding the optimal conditions for PCO/O_3_ process (Table 2). F-value and P-value in the analysis of variance (ANOVA) analysis are substantiated statistical indices for adjusting how to fit the data deviation factors. Because of these indexes, a significant statistical model with a high F-value and a low p-value (0.05), was selected. For the relationship between the predicted and experimental values of OTC and COD, Fisher's F-test indicated that a quadratic model

**Table 4:** An overview of the fit of the model.

Fit Summary of OTC						
Source	Sequential P-value	Adjusted R^2^	Predicted R^2^			
Linear	< 0.0001	0/6421	0/6133			
2FI	0/9883	0/5494	0/5111			
Quadratic	< 0.0001	0/9992	0/9975	Suggested		
Cubic	0/1723	0/9995	0/9815	Aliased		

Model summary statistics						
Source	Std. Dev	R^2^	Adjusted R^2^	Predicted R^2^	PRESS	
Linear	8/36	0/6914	0/6421	0/6133	2188/20	
2FI	9/38	0/7048	0/5494	0/5111	2766/75	
Quadratic	0/4060	0/9996	0/9992	0/9975	14/24	Suggested
Cubic	0/3224	0/9999	0/9995	0/9815	104/76	Aliased

Fit summary of COD						
Source	Sequential P-value	Adjusted R^2^	Predicted R^2^			
Linear	< 0.0001	0/6261	0/5954			
2FI	0/9888	0/5290	0/4859			
Quadratic	< 0.0001	0/9954	0/9863	Suggested		
Cubic	0/3015	0/9964	0/8744	Aliased		

Model summary statistics						
Source	Std. Dev	R^2^	Adjusted R^2^	Predicted R^2^	PRESS	
Linear	7/28	0/6777	0/6261	0/5954	1662/11	
2FI	8/17	0/6914	0/5290	0/4859	2111/89	
Quadratic	0/8062	0/9976	0/9954	0/9863	56/16	Suggested
Cubic	0/7155	0/9991	0/9964	0/8744	516/00	Aliased

would be the most appropriate fit (Table 4).

Using the ANOVA, the results of OTC and COD (Table 5) appeared highly reliable and had a very low probability value for the quadratic regression model, suggesting that it could accurately explain the codes within the actual data and predicted values. Based on the correlation coefficients (R^2^, R^2^adj, and R^2^ predict), this model proved exceptionally valid for predicting responses. There is a perfect correlation between R^2^, R^2^adj, and R^2^ predict, which suggests that the model has a high ability to predict responses^39,40^.

**Table 5:** ANOVA for a quadratic model in OTC, and COD in PCO/O_3_ process.

Source	OTC					COD					
	Sum of squares	df	Mean square	F-value	p-value	Sum of squares	df	Mean square	F-value	p-value	
Model	5656/18	14	404/01	2451/04	< 0.0001	4098/12	14	292/72	450/34	< 0.0001	Significant
A-pH	1356/01	1	1356/01	8226/53	< 0.0001	975/37	1	975/37	1500/58	< 0.0001	
B-Dose of MOFs	596/01	1	596/01	3615/81	< 0.0001	408/37	1	408/37	628/27	< 0.0001	
C-Reaction Time	1926/04	1	1926/04	11,684/78	< 0.0001	1365/04	1	1365/04	2100/06	< 0.0001	
D-O_3_ Concentration	34/56	1	34/56	209/67	< 0.0001	35/04	1	35/04	53/91	< 0.0001	
AB	0/3025	1	0/3025	1/84	0/1956	0/0625	1	0/0625	0/0962	0/7608	
AC	0/7225	1	0/7225	4/38	0/0537	0/5625	1	0/5625	0/8654	0/3670	
AD	6/25	1	6/25	37/92	< 0.0001	3/06	1	3/06	4/71	0/0464	
BC	8/70	1	8/70	52/80	< 0.0001	10/56	1	10/56	16/25	0/0011	
BD	3/24	1	3/24	19/66	0/0005	3/06	1	3/06	4/71	0/0464	
CD	56/25	1	56/25	341/25	< 0.0001	39/06	1	39/06	60/10	< 0.0001	
A^2^	243/78	1	243/78	1478/95	< 0.0001	181/57	1	181/57	279/35	< 0.0001	
B^2^	502/74	1	502/74	3050/00	< 0.0001	375/07	1	375/07	577/04	< 0.0001	
C^2^	237/69	1	237/69	1441/98	< 0.0001	181/57	1	181/57	279/35	< 0.0001	
D^2^	1228/97	1	1228/97	7455/85	< 0.0001	930/00	1	930/00	1430/77	< 0.0001	
**Residual**	2/47	15	0/1648			9/75	15	0/6500			
Lack of Fit	2/47	10	0/2472	2/8	0.03	9/75	10	0/9750	1/75	0.04	Not significant
Pure Error	0/0000	5	0/0000			0/0000	5	0/0000			
**Cor Total**	5658/65	29				4107/87	29				

The F-value of OTC, and COD is 2451/04, and 450/34 respectively. In the OTC and COD model, P-values of 0.0001 indicate a robust fit to the relationship between the experimental and predicted response values. A model discernment greater than 4.0 for the Adequate Precision ratio indicates a satisfactory model^41^. The polynomial equation ascribed to the OTC and COD removal (%) was represented in terms of coded factors:

22 $$ {\text{OTC}}\;{\text{removal}}\;\left( \% \right) = {9}0 + { 7}/{5} \times {\text{A}} + {4}/{9} \times {\text{B}} + {8}/{9} \times {\text{C}} + {1}/{2} \times {\text{D}} + 0/{13} \times {\text{AB}} + 0/{21} \times {\text{AC}} + 0/{6} \times {\text{AD}} - 0/{73} \times {\text{BC}} + 0/{4} \times {\text{BD}} - {1}/{8} \times {\text{CD}} - {2}/{9} \times {\text{A}}^{{2}} - {4}/{2} \times {\text{B}}^{2} - {2}/{9} \times {\text{C}}^{2} - {6}/{6} \times {\text{D}}^{2} $$

23 $$ {\text{COD}}\;{\text{removal}}\;\left( \% \right) = {72} + {6}/{3} \times {\text{A}} + {4}/{1} \times {\text{B}} + {7}/{5} \times {\text{C}} + {1}/{2} \times {\text{D}} - 0/0{6} \times {\text{AB }} + \, 0/{18} \times {\text{AC}} + 0/{4} \times {\text{AD}} - 0/{8} \times {\text{BC}} + 0/{4} \times {\text{BD}} - {1}/{5} \times {\text{CD}} - {2}/{5} \times {\text{A}}^{{2}} - {3}/{6} \times {\text{B}}^{{2}} - {2}/{5} \times {\text{C}}^{{2}} - {5}/{8} \times {\text{D}}^{{2}} $$

A positive and a negative effect of the studied mediators on the removal of OTC and COD is illustrated in Eqs. (22) and (23). The quadratic model had a low coefficient of variation (CV < 10%), which confirms the precision and reproducibility of the experiments. All interaction influences among all variables were significant. Based on the statistical analysis, the correlation between variables and the removal of OTC and COD was found to be statistically significant.

#### The impact of parameters on the OTC and COD removal on PCO/O_3_ process

**Figure 8:**
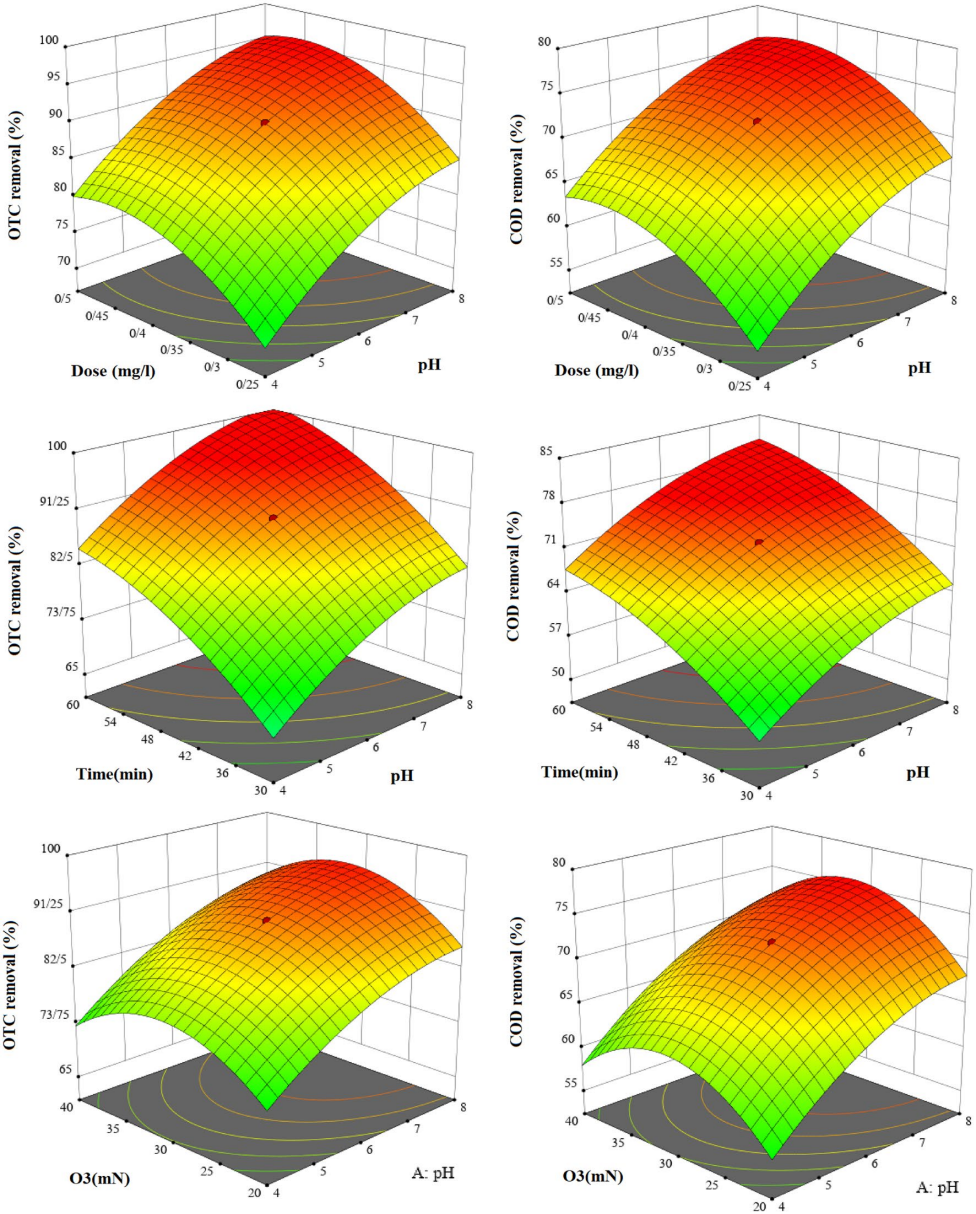
Effect of variables on removal of OTC and COD in PCO/O_3_ process.

PCO/O_3_ process highly depends on the pH of the solution, which has a substantial impact on the removal efficiency. Solution pH affects pollutant hydrolysis, ionization pollutants, catalyst surface characteristics, and activity rate of oxidants and reactive species, as well as the degradation route. Hence, the effects of pH (4–8) on OTC and COD removal efficiency were examined and illustrated in (Fig. 8). OTC and COD removal efficiency significantly increased with increasing solution pH, with pH of 8.0 achieving the highest removal efficiency. Electrostatic interactions between catalyst functional groups can be affected by pH (e.g., ionization) in this case. Also, when pH increases, ozone decomposes rapidly into radicals, which can degrade organic compounds much more rapidly and efficiently than ozone molecules^42^. It has been reported that OTC molecules have two pka values (3.18 as the strongest acidic and 8.29 as the strongest basic); therefore, OTC molecules are in cationic and anionic forms at the pka below 3.18 and above 8.29, respectively^43^. Meanwhile, the pH_zpc_ of the photocatalyst was 6.3, indicated that the photocatalyst surfaces act as protonates and non-protons in lower and higher, respectively. At lower pH levels, minimal adsorption was observed because of electrostatic repulsion between the positively charged catalyst and the protonated OTC molecules. Many studies have shown that contaminant decomposition occurs primarily through direct and indirect oxidation pathways in the ozonation process. OTC and ozone react directly at lower pH levels. As the pH of the solution was increased, ozone decomposition rate and free radical production increased, and free radicals with high oxidation potential were generated. These radicals demonstrate superior ability in OTC degradation. OTC molecules can react indirectly with free radicals in an alkaline environment, thus resulting in a higher removal efficiency. Asgari et al. investigated the ability of the photocatalytic ozonation process for ceftazidime removal. Based on this research, pH value at 11.0 was the optimum pH of solution^44^. The similar findings have been also reported by Espíndola et al.^45^. In this study, pH equal to 7.5 was reported as an optimum pH solution to oxytetracycline oxidation in the photocatalysis process.

##### Dose of photocatalyst

The dose of the photocatalyst is another important parameter that affected heterogeneous AOPs based on the catalyst. Different amounts of BiOI/MOF were applied to determine PCO/O_3_ efficiency in OTC removal (Fig. 8). Based on results, the removal efficiency of OTC was significantly enhanced by increasing the catalyst dose from 0.25 to 0.5 mg/l over 10 ppm of the initial concentration of OTC. Nevertheless, 0.4 mg/l catalyst doses did not show any notable differences. Higher catalyst concentrations had less favorable effects on the PCO/O_3_ process. Typically, this phenomenon is caused by particles accumulating and agglomerating and, reducing their surface active sites. Excessive catalyst dosages can scatter UV light in the solution, preventing the transmission of ultraviolet light and ozone to the catalyst surface. The surface area of the active site for photocatalytic activity can be increased by increasing catalyst content. As a result, a greater amount of ozone was adsorption on the surface of the nanoparticles, resulting in the production of more active radicals as a result of the breakdown of ozone. This improved OTC removal efficiency. Nanoparticles produce oxygen radicals when combined with ozone under alkaline conditions. When oxygen radicals are present in water, they create ^**·**^OH radicals, which eventually enhance ozonation efficiency^42^. Photocatalytic ozonation studies have reported similar findings, which agree with this study. Here, Lu et al. study tetracycline hydrochloride degradation by photocatalytic ozonation. In this research, zero to 0.8 g/l Bi_2_WO_6_ was applied as a catalyst. Until 0.5 g/l, performing process increased to 78% in 120 min then at 0.8 g/l the efficiency dropped to 65%^46^. Also in Yu et al. study, similar result obtained^47^.

##### Ozone concentration

Main important economic parameter in PCO/O_3_ process is ozone concentration. This parameter is closely related to energy consumption. An ozone gas concentration of 20–40 mM/l-min was investigated in this study (Fig. 8). The results showed that by increasing the concentration of ozone gas from 20 to 30 mN, the process's efficiency increased and decreased slightly. Higher ozone concentrations increase mass transfer in the reaction media. Concentrations of dissolved ozone in the solution increase as the ozone gas flow rate increases. Because of a synergistic effect, reactive oxygen species (ROS), particularly hydroxyl radicals, are produced in greater quantities. Photocatalyst easily adsorb dissolved ozone molecules because of weak hydrogen bonds with their surface ^**·**^OH groups. Anions of ozonide radicals are produced by capturing electrons on the surface of the photocatalyst. Thus, the ozonide radicals (O_3_^**·**−^) increase the amount of ^**·**^OH radicals and the efficacy of OTC degradation^46^. When incoming air flow increases excessively, and the mass transfer rate from the gas phase to the liquid phase is limited, the efficacy of removing decreases. The concentration of ozone required for PCO/O_3_ depends on the type of process, the type of reaction reactor, the type of pollutant, and the specifications of the intermediate compounds^48^. In previous studies, a different concentration of ozone was reported as an optimum concentration. Yu et al.^49^ 1.5 mg/l-min of ozone concentration, Heydari et al.^50^ 11 mg/l of ozone concentration was reported as an optimum ozone concentration.

##### Reaction time

The reaction time is another important parameter that affected heterogeneous AOPs. In the current study, the effect of reaction rime on PCO/O_3_ effect in OTC and COD removal in 30 to 60 min range was investigated (Fig. 8). Based on (Fig. 0d), significant increase in degradation efficiency is observed for both OTC and COD with increasing reaction time. As mentioned, PCO/O_3_ process removes OTC through two direct and indirect oxidation mechanisms. Direct oxidation by ozone molecules and photolysis and indirect oxidation based on free radicals and electron–hole pairs produced cause pollutant destruction. So increasing the reaction time allowed for complete pollutant destruction and mineralization, and less intermediate compounds formed. Therefore, the optimal reaction time depends on the type of pollutant, characteristics of the reaction reactor, other operating parameters, and operating conditions. For this reason, different times have been reported in previous studies. Lu et al.^46^, report 120 min as the optimal time.

Finally, we used Design-Expert software to estimate the optimal value of experiment variables for OTC degradation by PCO/O_3_. pH of solution 8.0, dose of catalyst 0.34 mg/l, reaction time 56 min and ozone concentration 28.7 mN at 10 mg/l as an initial concentration of OTC were an optimum condition of PCO/O_3_. In this situation, 96.2, and 77.2% of OTC and COD were removed, respectively.

#### Synergist effect, TOC removal and recyclability of photocatalyst

**Figure 9:**
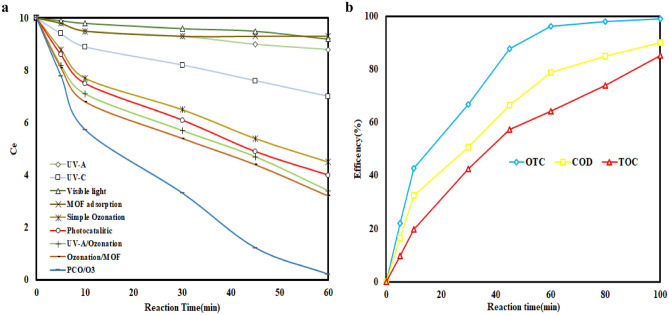
(**a**) Synergistic effect in PCO/O_3_ process. (**b**) OTC, COD and TOC removal in PCO/O_3_ process in optimum condition (pH 8.0, DOC = 0.34 mg/l, RT = 60 min, O3 = 28.7 mN, OTC = 10 mg/l).

Figure 9a shows the results of comparing the roles of the single, binary, and PCO/O_3_ processes in the degradation of OTC under optimal conditions.

**Figure 10:**
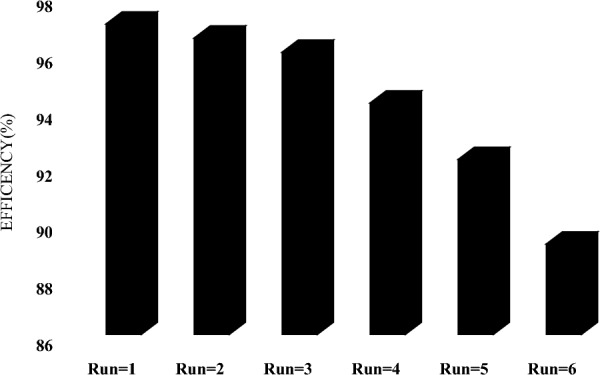
Catalyst recycling test in PCO/O_3_ process (pH 8.0, DOC = 0.34 mg/l, RT = 60 min, O_3_ = 28.7 mN, OTC = 10 mg/l).

According to the results, simple ozonation (55%), photolysis (UV-C = 30%, UV-A = 12, and visible light = 8%), and sorption (7%) are ineffective in OTC degradation. Therefore, these mechanisms do not have satisfactory potential for degrading OTC individually. O_3_/catalyst (68%), UV/ catalyst (60%), and UV/O_3_ (66%) as binary processes exhibited more efficient OTC degradation than the individual processes. Ultimately, over 95% of the tetracycline concentration was removed through the fundamental process. This study confirms a synergistic effect between various processes, resulting in a more active degradation of OTC and an excess creation of super reactive radicals. However, when all variables were involved simultaneously, OTC removal performance significantly improved. Based on (Eq. 20), the SF coefficient of 1.31 was obtained. When light irradiates to catalyst, electrons are produced in the conduct band (CB) and holes are formed in the valance band (VB). Since the catalyst's CB potential is higher than the redox potential of oxygen/oxygen peroxide in the reaction medium, electrons transfer to the oxygen in the reaction media and are converted into oxygen abundantly. The photocatalytic system will be affected by the utilization of the photo-generated carriers and the production of ROS. In contrast to its direct ozonation, electrophilic ozone easily traps photo-generated electrons when introduced into photocatalytic systems. Because of the effective transfer of the photo-generated electron of catalyst, the electron–hole pairs are efficiently separated under simulated light irradiation, leading to a large amount of ROS being produced. A catalyst can also improve the O_3_/Light system's ozone utilization ratio. In the reaction, more ozone will produce more ROS, which is beneficial to mineralization in removing organic compounds. It is possible to remove organic pollutants effectively by combining photocatalysis with ozonation, as the combined process produces more ROS than the sum of the individual processes. In Lu et al.^46^ study, TOC removal in O_3_, O_3_/light and O_3_/light/catalyst were 18, 20 and 78% respectively. In (Fig. 9b) the results of OTC, COD, and TOC removal in 0–100 min reaction time presented. According to the results, it has been found that the COD and TOC removal rate is lower than OTC. Under optimal conditions, OTC, COD, and TOC decreased by 96.2, 77.2, and 64.2%, respectively. The existing difference indicates the formation of organic intermediates and by-products. Also, the results showed that increasing the reaction time increases the efficiency of removing COD and TOC and more mineralization. Asgari et al.^42^ reported 100, 92.3 and 81.1% CIP, COD and TOC removal respectively, in 60 min. Stability and recyclability are essential characteristics of catalysts for real applications. As a result, photocatalyst recyclability was investigated over six consecutive cycles under optimal conditions. After each cycle, the catalyst was separated from the solution and dried for 24 h at 80 °C. According to (Fig. 10), OTC removal efficiency decreased from 97 to 89.2% after six consecutive cycles. Photocatalytic ozonation efficiency decreased slightly (i.e., by 8%) suggesting that catalysts can be reused for at least six consecutive cycles. gradual loss of photocatalytic adsorption sites as a result of blockages caused by OTC and intermediates, Utilization of active oxidizing species by intermediates and Continuous washing and drying reduce the number of active photocatalytic sites are reasons explaining the slight decrease in performing catalyst^44^. Bagheri et al.^51^, 92% efficiency in dye degradation reported after eight repetitive cycles of catalyst.

#### Organic scavengers and co-existing ions effect

**Figure 11:**
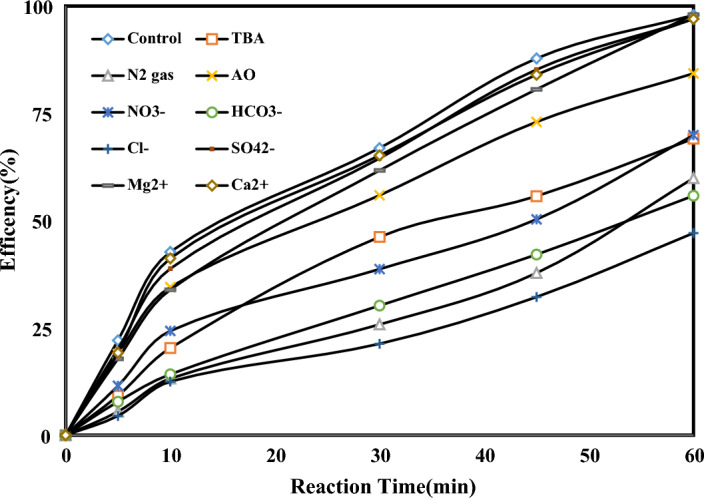
Effect of ions and radical scavengers on OTC removal in PCO/O_3_ process (pH 8.0, DOC = 0.34 mg/l, RT = 60 min, O_3_ = 28.7 mN, OTC = 10 mg/l).

Many types of inorganic ions are found in practical wastewater. It is important to study how different inorganic ions can affect PCO/O_3_ ability to remove pollutants. The influence of anions and cations on the PCO/O_3_ performance is illustrated in (Fig. 11). Nitrate (NO_3_^−^ = 0.1 mM), Bicarbonate (HCO_3_^−^ = 0.1 mM), and Chloride (Cl^−^ = 0.1 mM) had inhibited PCO/O_3_ process efficiency. However, the PCO/O_3_ process was not affected by adding sulfate (SO_4_^2−^). NO_3_^−^, HCO_3_^−^ and Cl^−^ ions use the ROS in the PCO/O_3_ process. Cl^−^ ion readily adsorbs on the catalyst surface, preventing reactant molecules from adhering to it, reducing the reaction's efficiency. The process is not affected by the addition of Na^+^ or K^+^. A solution containing Mg^2+^ or Ca^2+^ promotes the removal of OTC. In Lu et al.^46^ and Asgari et al.^44^ similar finding reported. OTC removal was investigated using scavenging tests over PCO/O_3_ process to identify reactive oxidizing species. Free radical species (O_2_^**·**−^, O_2_^1^, OH^**·**^, e^-^—h^+^) were evaluated by quenching tests for their effect on removing OTC^52^. OA acts as a scavenger of O_2_^**·**-^ and e^-^—h^+^ pairs. TBA is the scavenger of O_2_^1^ and OH^**·**^, and finally N_2_ gas is a scavenger of all ROS_s_. Based on results indicated that, performing PCO/O_3_ process in the presence of AO, it drops less, in the presence of TBA, the rate of decrease in process efficiency is higher, and N_2_ gas causes a significant decrease in efficiency. Therefore, O_2_^1^ and OH^**·**^ were the main active radicals. Similar finding reported in previous studies^48,53^.

#### Kinetic of reaction and electrical energy consumption

**Figure 12:**
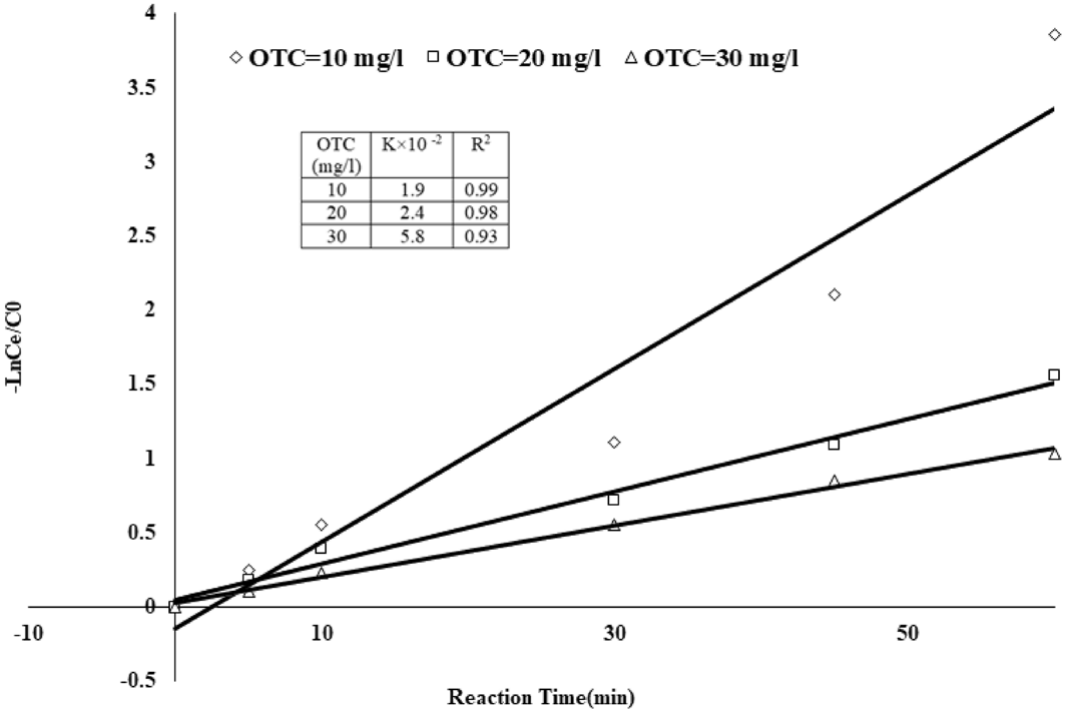
OTC degradation kinetics in PCO/O_3_ process (pH 8.0, DOC = 0.34 mg/l, RT = 60 min, O_3_ = 28.7 mN, OTC = 10, 20 and 30 mg/l).

On optimized conditions, the pseudo-first-order model (Eq. 19) described the OTC degradation rate over the PCO/O_3_ process. As shown in (Fig. 12), OTC degrades over PCO/O_3_ in 10, 20, and 30 mg/l concentrations over a 60 min reaction using a linear pseudo-first-order kinetic model. According to the pseudo-first-order model, OTC degradation kinetics in PCO/O3 are well supported by the experimental data (R2 > 0.99). Based on the review of previous studies, it was found that in most AOPs, the reaction kinetics follows pseudo-first-order kinetics^54,55^.

In practical applications, energy consumption must be considered. The ozone generator and the UV lamp handled EEC in this process. The total energy consumption of was 220 W. According to (Eq. 21), EEC of PCO/O_3_ process under optimum condition was 4.44 KWh. Based on previous studies, the EEC depends on various factors such as the power of the ozone generator, the power of the lamp, the reaction time, the volume of the reaction chamber and finally the efficiency of the process^56^. In Kang et al. study, 55 KWh/m^3^ reported as EEC^10^.

#### Identifying the mineralization pathway for OTC

**Figure 13:**
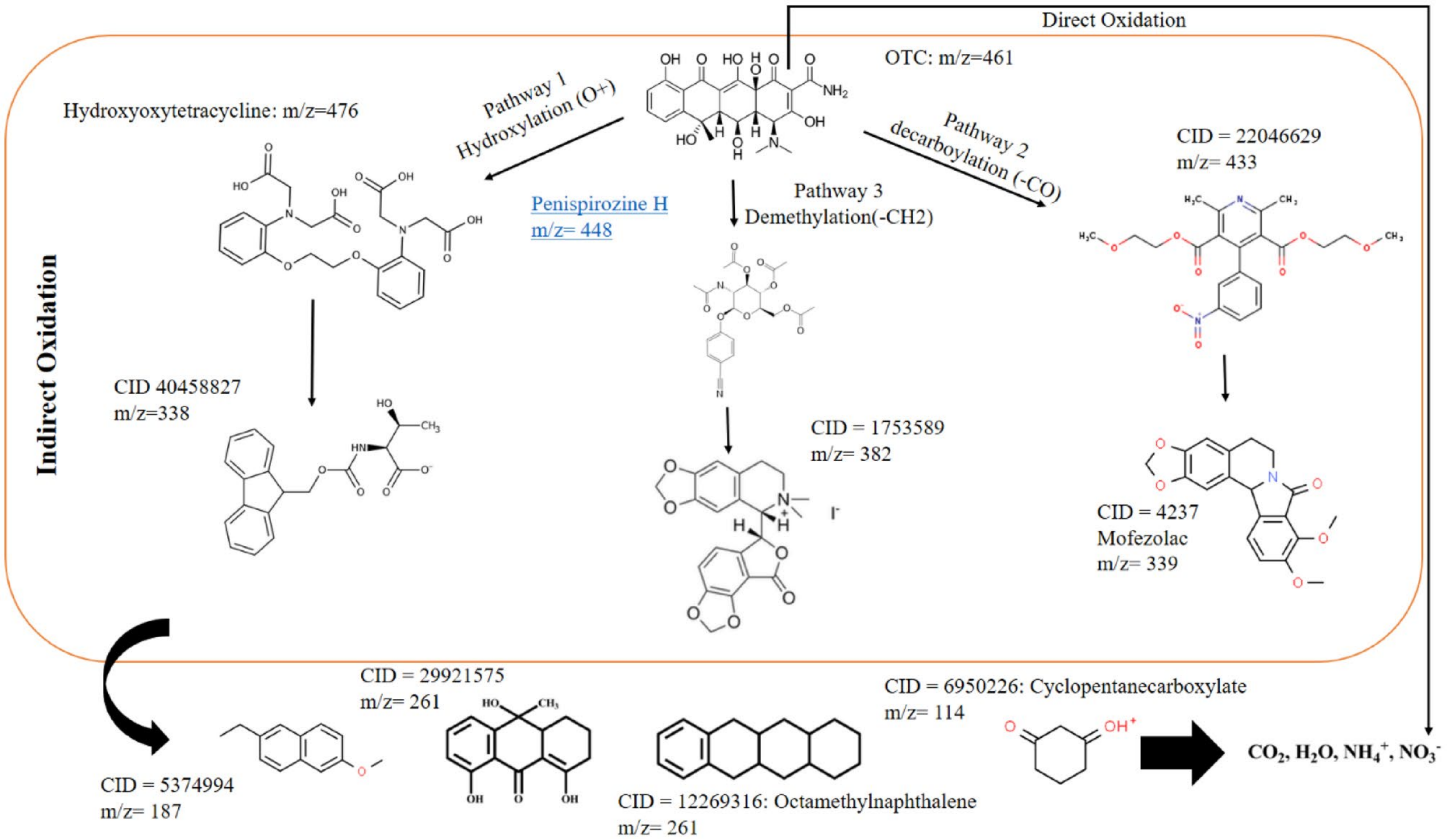
Proposed degradation pathway of OTC by the PCO/O_3_ process in optimum condition (pH 8.0, DOC = 0.34 mg/l, RT = 60 min, O_3_ = 28.7 mN, OTC = 10 mg/l).

To identify the intermediates produced during OTC degradation, GC–MS experiments were conducted to reveal the degradation pathway. According to the results analysis and previous relative studies, two main pathways were identified for OTC degradation: direct oxidation and indirect oxidation. During direct oxidation, mineralization plays a minimal role. Indirect oxidation destroys OTC through hydroxylation, demethylation, and decarbonylation (Fig. 13). OTC's aromatic ring is attacked by hydroxyl radicals (pathway 1) through the reaction of the OH addition. By-product P1 had been formed after OH attacked the keto-Enol at C11a–C12 (m/z 476). As a result of the decarbonylation (pathway 2), OTC loses CO from its ring structure, which produces the byproduct P6 (m/z 433). In the chemical decomposition of acetylacetone, CO was generated by the rapid dissociation of COCH_3_ formed by the cyclic cleavage of enolic acetylacetone. At the C4 target site, one methyl group of dimethylammonium group might be eliminated, resulting in intermediate P3 (m/z 448)^57^. By review of previous studies, it was found that based on the process used, different intermediate compounds and different reaction paths may occur in the degradation of OTC.

To conclude, Table 6 demonstrates multiple studies that assessed different AOPs strategies for removing OTCs from aquatic solutions.

**Table 6:** Different AOPs studies in OTC removal.

Processes	Method	Efficiency	Refs.
Cu-SACs/peroxymonosulfate	Peroxymonosulfate, catalyst dose, and initial pH were investigated	96.47% at 60 min	^58^
plasma-catalytic/graphene-TiO_2_-Fe_3_O_4_ nanocomposites	catalyst dosage, peak voltage, air flow rate and pH value were investigated	98.1%	^59^
Electrochemical/Ti/IrO_2_-Ta_2_O_5_ anode	Electrical current, plate spacing, electrolyte concentration, and solution pH were investigated	99.02% at 20 min	^60^
Fenton-like	rGO in Pd/nZVI/rGO composite rateio, initial concentration of OTC, reaction time, and pH were investigated	96.5% at 60 min	^61^
UV/peracetic acid	pH and a PAA dose were investigated	completely removed in 45 min	
PCO/O_3_	pH, reaction time, O_3_ concentration, and catalyst dose were investigated at 10 mg/l OTC concentration	96.2% removed at 60 min	Current study

## Conclusion

In this study, BiOI-MOF was synthesized by solvo-thermal method. Based on results of XRD, FESEM, EDAX, FTIR, UV–Vis spectra, TEM, BET, and XPS analysis indicated that synthesis of BiOI/MOF was done with best character. Design of experiment, ANOVA statistical analysis, interaction of parameters and predicated optimum condition was done based on CCD. The effect of pH of solution (4–8), catalyst dose (0.25–0.5 mg/l), reaction time (30–60 min) and O_3_ concentration (20–40 mN) at 10 mg/l of OTC on PCO/O_3_ process optimized. Based on F-value and P-value coefficients, the model of OTC (F-value = 2451.04, P-value = 0.0001) and COD (F-value = 450.3, P-value = 0.0001) removal was quadratic. Under optimum condition pH 8.0, CD = 0.34 mg/l, RT = 56 min and O3 concentration = 28.7 mN, 96.2 and 77.2% of OTC and COD removed, respectively. TOC decreasing under optimum condition was 64.2% that lower than OTC and COD. The kinetic of reaction follows pseudo-first-order kinetics (R^2^ = 0.99). Synergistic effect coefficient was 1.31 that indicated ozonation, presence of catalyst and photolysis had a synergistic effect on OTC removal. The stability and reusability of catalyst in six consecutive operating was acceptable and 7% efficiency decreased only. Cations (Mg, and Ca), SO_4_^2−^ had no effect on the efficiency of the process, but other anions, organic scavengers and N_2_ gas, had an inhibitory effect on the efficiency of the process. EEC under optimum condition 4.44 KWh/m^3^ calculated. Finally, the OTC degradation probably pathway includes direct and indirect oxidation that Hydroxylation, Demethylation, and Decarboxylation were the fundamental mechanism in OTC degradation.

## Data Availability

The datasets generated and analyzed during the current study were available from the corresponding author on reasonable request.
